# Cough peak expiratory flow. assistance at extubation and outcomes

**DOI:** 10.1186/2197-425X-3-S1-A664

**Published:** 2015-10-01

**Authors:** P Carmona Sanchez, J Muñoz Trujillo, MD Bautista Rodriguez, V Martinez de Pinillos Sanchez, M Echeverria Leon, R Diaz Pernalete, I Durban Garcia, JM Serrano Simon

**Affiliations:** Hospital Universitario Reina Sofia, Intensive Care Unit, Cordoba, Spain

## Introduction

Cough strength predict extubation outcomes of patients who have passed a spontaneous breathing trial (SBT) (1,2).

## Objectives

To evaluate the impact on extubation outcome of prophylactic noninvasive assistance at extubation of patients with weak cough, and to identify optimal device for assistance.

## Methods

Prospective collected database was conducted from December 2014 to April 2015. Weak cough was defined by peak expiratory flow (PEF) < 60 L/min. The PEF was measured with Cosmed Pony Graphic^®^ spirometer v.4.0 S-CZ before extubation, for the patients mechanically ventilated >24 h, who passed successfully SBT at least of 30 min of pressure support 5-8 cmH2O, CPAP, or T-T. The patients were then extubated regardless the PEF. The patients with PEF>60 L/min conventional oxygen therapy was applied, and groups at risk of extubation failure (PEF< 60 L/min) was applied prophylactic noninvasive ventilation (vni) [BiLEVEL/CPAP mode] vs humidified high-flow nasal cannula (HFNC) randomly. Extubation failure was defined as the need of reintubation within 48 h following extubation. We compared both groups of patients according to the PEF, and prophylactic assistance on groups patients with weak cough, on outcome extubation. Continuous variables were expressed as mean ± SD or median (IRQ) and categorical variables as absolute value and percentage. The comparison of continuous variables was performed by Student t test and Mann-Whitney test and comparison between categorical variables was performed by Fisher's exact test and Chi-square test.

## Results

Sixty-six patients were studied, 48 males (72,7%). The two groups of patients according to the PEF, were similar regarding age, APACHE II, underlying chronic disease and duration of mechanical ventilation before extubation. Prophylatic assistance was effectively applied to 78,3% of patients identified at risk of extubation failure. Vni was applied at 43,5% (CPAP 17,4% and BiLevel 26,1%), HFNC 56,5%. No significant differences were found between the prophylactic devices of assistance at risk group of extubation failure. In the patients with PEF>60 L/min, extubation failure rate was 16.3% The higher proportion of extubation failure were in the neurological patients (21,2%). In this group of patients, the PEF<60 L/min were 27,8%. There were no differences in ICU admission days in both groups considering the PEF.

## Conclusions

Prophylactic assistance to the patients identified with a weak cough strength at extubation could reduce the risk of extubation failure. The application of prophylactic assistance at extubation, also could be beneficial in some patients with PEF>60 L/min and to reduce extubation failure. The prophylactic assistance can be applied to any device and mode (vni or HFNC)
